# Adherence and invasive properties of *Corynebacterium diphtheriae* strains correlates with the predicted membrane-associated and secreted proteome

**DOI:** 10.1186/s12864-015-1980-8

**Published:** 2015-10-09

**Authors:** Vartul Sangal, Jochen Blom, Iain C. Sutcliffe, Christina von Hunolstein, Andreas Burkovski, Paul A. Hoskisson

**Affiliations:** Faculty of Health and Life Sciences, Northumbria University, Newcastle upon Tyne, NE1 8ST UK; Heinrich-Buff-Ring 58, Justus-Liebig-Universität, 35392 Gießen, Germany; Istituto Superiore di Sanità, viale Regina Elena 299, 00161 Rome, Italy; Professur für Mikrobiologie, Friedrich-Alexander-Universität Erlangen-Nürnberg, Staudtstr. 5, 91058 Erlangen, Germany; Strathclyde Institute of Pharmacy and Biomedical Sciences, University of Strathclyde, 161 Cathedral Street, Glasgow, G4 0RE UK

**Keywords:** *Corynebacterium diphtheriae*, Whole genome sequencing, Virulence, Secretome, Proteome

## Abstract

**Background:**

Non-toxigenic *Corynebacterium diphtheriae* strains are emerging as a major cause of severe pharyngitis and tonsillitis as well as invasive diseases such as endocarditis, septic arthritis, splenic abscesses and osteomyelitis. *C. diphtheriae* strains have been reported to vary in their ability to adhere and invade different cell lines. To identify the genetic basis of variation in the degrees of pathogenicity, we sequenced the genomes of four strains of *C. diphtheriae* (ISS 3319, ISS 4060, ISS 4746 and ISS 4749) that are well characterised in terms of their ability to adhere and invade mammalian cells.

**Results:**

Comparative analyses of 20 *C. diphtheriae* genome sequences, including 16 publicly available genomes, revealed a pan-genome comprising 3,989 protein coding sequences that include 1,625 core genes and 2,364 accessory genes. Most of the genomic variation between these strains relates to uncharacterised genes encoding hypothetical proteins or transposases. Further analyses of protein sequences using an array of bioinformatic tools predicted most of the accessory proteome to be located in the cytoplasm. The membrane-associated and secreted proteins are generally involved in adhesion and virulence characteristics. The genes encoding membrane-associated proteins, especially the number and organisation of the pilus gene clusters (*spa*) including the number of genes encoding surface proteins with LPXTG motifs differed between different strains. Other variations were among the genes encoding extracellular proteins, especially substrate binding proteins of different functional classes of ABC transport systems and ‘non-classical’ secreted proteins.

**Conclusions:**

The structure and organisation of the *spa* gene clusters correlates with differences in the ability of *C. diphtheriae* strains to adhere and invade the host cells. Furthermore, differences in the number of genes encoding membrane-associated proteins, e.g., additional proteins with LPXTG motifs could also result in variation in the adhesive properties between different strains. The variation in the secreted proteome may be associated with the degree of pathogenesis. While the role of the ‘non-classical’ secretome in virulence remains unclear, differences in the substrate binding proteins of various ABC transport systems and cytoplasmic proteins potentially suggest strain variation in nutritional requirements or a differential ability to utilize various carbon sources.

**Electronic supplementary material:**

The online version of this article (doi:10.1186/s12864-015-1980-8) contains supplementary material, which is available to authorised users.

## Background

Diphtheria is a toxin mediated infection of upper-respiratory tract which is caused by *Corynebacterium diphtheriae*. The global immunisation programme using diphtheria-toxoid has significantly reduced the burden of disease [1]. The major virulence factor, diphtheria toxin, is encoded by the *tox* gene on the lysogenic β-corynephage [2, 3]. The diphtheria vaccine is only effective against toxigenic strains and the number of infections caused by non-toxigenic *C. diphtheriae* reported worldwide is increasing [4–7]. An infection by a non-toxigenic *C. diphtheriae* strain may vary from a localized respiratory tract infection to more invasive endocarditis, septic arthritis and osteomyelitis [8–12]. The severity of the disease is dependent on the ability of *C. diphtheriae* strains to adhere to host cells, intracellular viability and the induction of cytokine production by the host immune system [13–16].

Non-toxigenic *C. diphtheriae* strains have been shown to significantly vary in their macromolecular surface structure and cell adhesion properties, although, no clear correlation between pili formation, adhesion and invasion could be established [17]. The differences in the structure of pili correlated with the strain specific variations in the expression of pili subunits [17]. Comparative genomic analyses revealed that the pilus gene clusters are borne on horizontally acquired pathogenicity islands and the number of these clusters and the genes encoding the subunits of adhesive pili within each cluster varied among *C. diphtheriae* strains [18].

Bacterial cell surface and extracellular proteins play important roles as the interface between host and pathogen and represent key virulence characteristics [19–21] but little is known about the roles of these proteins in pathogenesis by *C. diphtheriae*, beyond the extensively studied secreted toxin [22, 23]. Our previous analyses of 17 *C. diphtheriae* genomes revealed differences in the gene content between different strains [24, 25]. However, a large proportion of genes in these genomes encode hypothetical or uncharacterised proteins, which is a major hindrance in inferring the protein function and potential involvement in virulence. In this study, we have sequenced the genomes of an additional four non-toxigenic *C. diphtheriae* strains with varying degrees of virulence potential that has been previously assayed experimentally and compared the sequences to identify the gene content that maybe associated with the variation in disease severity. We have also analysed and categorised the genome-wide predicted proteome of all publicly available *C. diphtheriae* genomes to identify genes encoding membrane-associated and secreted proteins that may play a role in host-pathogen interaction.

## Results

### Genomes of invasive non-toxigenic *C. diphtheriae*

The non-toxigenic *C. diphtheriae* strains ISS 3319, ISS 4060, ISS 4746 and ISS 4749 were isolated from patients with severe pharyngitis and tonsillitis in Italy [4, 15]. In an infection model, ISS 4746 and ISS 4749 caused relatively severe arthritis in a higher proportion of mice and induced higher levels of interleukin (IL)-6 and IL-1*β* in comparison to ISS 3319 [15]. The genomes of strains ISS 3319, ISS 4060, ISS 4746 and ISS 4749 were sequenced on a GS Junior instrument (Roche) and were assembled into 33 contigs, 47 contigs, 60 contigs and 40 contigs, respectively. The sizes of the whole genome assemblies and the number of genes were comparable among the four *C. diphtheriae* strains as summarised in Table 1. The genome sequences of these strains are available from the GenBank with the accession numbers JAQO00000000, JAQN00000000, JAQP00000000 and JAQQ00000000, respectively.

**Table 1:** Details of the genome assemblies of *C. diphtheriae* strains ISS 3319, ISS 4060, ISS 4746 and ISS 4749

Details	ISS 3319	ISS 4060	ISS 4746	ISS 4749
Number of reads	218,328	188,708	164,106	186,176
Average read length (bp)	479	463	454	467
Size of assembly (bp)	2,366,093	2,395,664	2,379,713	2,366,484
Number of contigs	33	47	53	40
Average coverage	70x	58x	55x	66x
N50 (bp)	139,564	102,641	119,602	145,623
Average GC content (mol%)	53.53	53.61	53.46	53.45
Number of features	2,272	2,284	2,275	2,258
- CDS	2,168	2,198	2,185	2,168
- rRNA	3	4	4	2
- tRNA	49	46	52	49
- ncRNA	1	1	1	1

### Variation in the gene content among invasive non-toxigenic *C. diphtheriae*

**Fig. 1:**
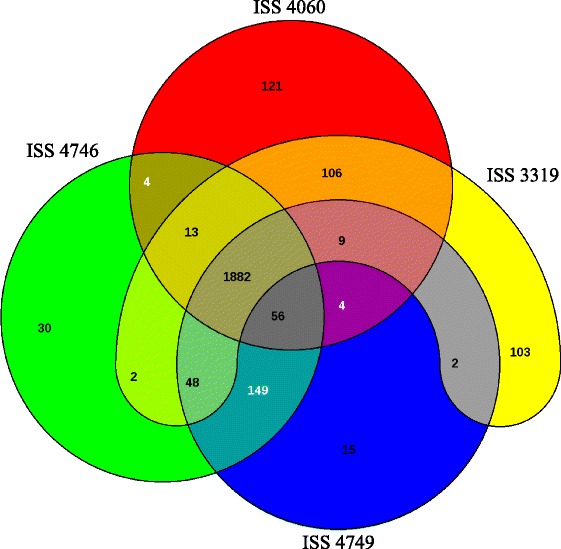
A Venn diagram showing the numbers of shared and specific genes among *C. diphtheriae* strains ISS 3319, ISS 4060, ISS 4746 and ISS 4749

Whole genome sequences of these strains were compared using EDGAR [26], excluding the genes with partial sequences due to gaps in the assembly. Most of the genes (1,882 genes) were shared among the four strains but a small proportion of genes were not present in all the four strains (Fig. 1). Specific gene content comprised 103 and 121 genes in ISS 3319 and ISS 4060, respectively which is relatively high compared to the number of genes restricted to ISS 4746 (30 genes) and ISS 4749 (15 genes). More than 97.8 % of the genes (2,135 genes) in ISS 4746 (2,184 genes) and ISS 4749 (2,165 genes) were shared with each other. Indeed, these two strains belong to the same sequence type (ST), ST32, which is distinct from ISS 3319 (ST26) and ISS 4060 (ST5).

**Fig. 2:**
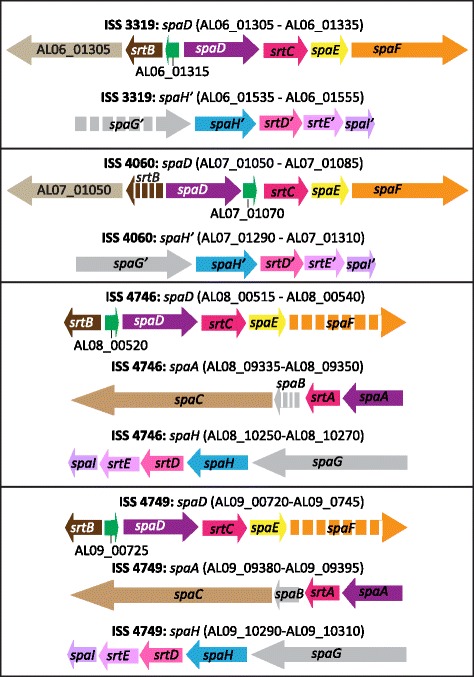
Structure and organisation of pilus gene clusters in *C. diphtheriae* strains ISS 3319, ISS 4060, ISS 4746 and ISS 4749. The common genes between different strains are shown in the same colour and the pseudogenes are shown using broken arrows. The direction of the arrow indicates the orientation of the coding sequence. The schematic is not to scale

Most of the strain specific accessory genes (not present in all four strains) encode hypothetical proteins (Table 2, Additional file 1: Table S1). Since ISS 4746 and ISS 4749 belong to the same ST, 149 genes shared by them but not with ISS 3319 and ISS 4060 were also considered to be strain-specific. A fraction of the strain specific genes encode fimbrial sub-units or membrane associated proteins including the genes belonging to the pilus gene clusters (Table 2; Fig. 2). These gene clusters encode adhesive pili that are important for adhesion and invasion of the host cells [27–29]. All the four strains were found to carry the SpaD and SpaH pilus gene clusters; however, an additional gene cluster, SpaA, was present in strains ISS 4746 and ISS 4749 (Fig. 2). The number and organisation of genes in the SpaD cluster was found to vary. An additional gene encoding a putative surface-anchored fimbrial subunit was present in strains ISS 3319 and ISS 4060. A gene encoding a hypothetical protein was present between the *srtB* and *spaD* genes in strains ISS 3319, ISS 4746 and ISS 4749, and between *spaD* and *srtC* in ISS 4060 (Fig. 2). In addition, *srtB* in ISS 4060 and *spaF* in ISS 4746 and ISS 4749 were found to be pseudogenes. Similarly, the *spaG* gene in the SpaH cluster of ISS 3319 and the *spaB* gene in the SpaA cluster of ISS 4746 were found to be inactive. This variation in the number of pilus genes in each cluster is in agreement with previously observed variations in the macromolecular surface structures and the expression of *spa* genes among these strains [17]. These results are also consistent with Trost et al. who reported variation in the Spa clusters in *C. diphtheriae* [18].

**Table 2:** Strain-specific gene content based on the comparison of the draft genomes of *C. diphtheriae* strains ISS 3319, ISS 4060, ISS 4746 and ISS 4749

Protein annotation	ISS 3319	ISS 4060	ISS 4746	ISS 4749	ISS 4746 – ISS 4749^a^
Hypothetical	69	73	22	10	86
Fimbrial & membrane	4	4	-	2	12
Transposase	6	13	3	1	14
Others^b^	24	31	5	2	37

^a^Strains ISS 4746 and ISS 4749 belong to the same ST, ST32. Hence, the genes shared by them but not with ISS 3319 and ISS 4060 were treated as strain-specific and these are detailed in the column “ISS 4746 – ISS 4749”

^b^CDS encoding transcription regulators, components of restriction-modification systems or other cellular and metabolic activities were grouped within the ‘Others’ category

Other strain specific genes are summarised in Additional file 1: Table S1 and include transposases and gene involved in metabolism, transcription, DNA replication, transport function and defence mechanisms. These genes do not appear to have a clear role in pathogenesis. However, a number of strain specific proteins are hypothetical proteins with uncharacterised functions and may yet be shown to contribute to pathogenicity in *C. diphtheriae* strains.

### Core and accessory proteome of *C. diphtheriae*

A proper identification of gene function is the key to understanding the cellular and virulence mechanisms which is dependent on the accuracy of genome annotation. A major hindrance in functional inference from genome analysis is the large proportion of annotated hypothetical proteins, uncharacterised proteins or proteins of unknown function [30]. The majority of variation between invasive *C. diphtheriae* strains in this analysis is hypothetical proteins, similar to our previous study [25]. Therefore, we analysed the entire genome content within *C. diphtheriae* including 16 previously published genomes (Additional file 2: Table S2) [18, 24, 25, 31–33] using a range of programs to identify cellular, membrane-associated and secreted proteins to gain a greater understanding of these hypothetical proteins and to investigate correlations between strains exhibiting various degrees of pathogenicity.

**Fig. 3:**
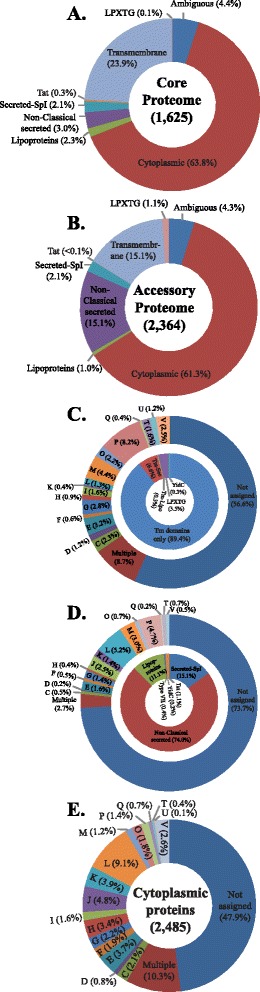
The distribution of predicted protein functions (**a**) in the core genome, (**b**) in the accessory genome of *C. diphtheriae*. The distribution of the functional protein categories based on the similarities in the Clusters of Orthologous Genes (COG) database within predicted (**c**). Transmembrane proteins (outer circle), (**d**) secreted proteins (outer circle) and (**e**) cytoplasmic proteins. The proportion of different types of membrane-associated and secreted proteins is shown in the inner circles within panel **c** and **d**, respectively. (COG codes - A: RNA processing and modification; B: Chromatin structure and dynamics; C: Energy production and conversion; D: Cell cycle control, cell division, chromosome partitioning; E: Amino acid transport and metabolism; F: Nucleotide transport and metabolism; G: Carbohydrate transport and metabolism; H: Coenzyme transport and metabolism; I: Lipid transport and metabolism; J: Translation, ribosomal structure and biogenesis; K: Transcription; L: Replication, recombination and repair; M: Cell wall/membrane/envelope biogenesis; N: Cell motility; O: Post-translational modification, protein turnover, and chaperones; P: Inorganic ion transport and metabolism; Q: Secondary metabolites biosynthesis, transport, and catabolism; T: Signal transduction mechanisms; U: Intracellular trafficking, secretion, and vesicular transport; V: Defence mechanisms; W: Extracellular structures; Y: Nuclear structure; Z: Cytoskeleton; Not assigned: General function prediction and unknown function)

The pan-genome of 20 *C. diphtheriae* strains was calculated using EDGAR [26] and was found to be comprised of 3,989 genes including 1,625 core genes and 2,364 accessory genes (Additional file 3: Table S3). The total predicted core and accessory proteome of *C. diphtheriae* was further analysed to determine potential functional localisation. The majority of the core proteins (1,037/1,625 proteins) were predicted to be cytoplasmic (Fig. 3a; Additional file 3: Table S3). 390 core proteins (24 %) were predicted to be transmembrane proteins, including 21 with a signal peptide. A single transmembrane domain was detected in 72 proteins and 310 protein sequences had two or more transmembrane domains. The remaining eight of these proteins were either predicted to have one transmembrane domain by Phobius [34] but two by TMHMM 2.0 [35] or vice versa (Additional file 3: Table S3). Transmembrane proteins with a single domain might be peripheral or associated with cell envelope while those with multiple domains are predicted integral membrane proteins.

One hundred twenty-seven proteins were predicted to be secreted proteins, including 37 (29.1 %) lipoproteins and 49 (42.2 %) non-classical secreted proteins (Additional file 3: Table S3). Non-classical secreted proteins do not have signal peptides but are known to be extracellular and secreted via Sec-independent pathways [36]. Two proteins were identified as substrates for type VII secretion systems and five core proteins were predicted to be secreted through the Twin-Arginine Translocation (Tat) secretion pathway (Fig. 3a; Additional file 3: Table S3). The remaining 71 proteins were considered ambiguous in that they could not be assigned to any specific location due to a lack of consensus between different prediction programs (as described in Methods).

Similar to the core proteome, 61.3 % of the accessory genome was predicted to encode cytoplasmic proteins (Fig. 3b; Additional file 3: Table S3). Additional variation between *C. diphtheriae* genomes was among the transmembrane (16.2 %; including 26 proteins with a LPXTG domain) and secreted proteins (18.1 %). Approximately 82.9 % of the secreted proteins were predicted to be non-classical secreted proteins. Membrane-associated and secreted proteins play crucial roles in pathogenesis [37–39] and the variation in these proteins between different strains might therefore be associated with varying virulence. The accessory proteome also included 102 proteins that were scored as ambiguous regarding their localisation, as described above (Additional file 3: Table S3).

### Proteins with predicted transmembrane domains

Transmembrane domains were identified in 772 proteins that were predicted to be involved in a variety of functions including inorganic ion transport and metabolism (8.2 %) and defence mechanisms (2.5 %; Fig. 3c; Additional file 3: Table S3). Sixty-seven of these proteins were assigned to multiple COG categories while no category was predicted for 437 proteins (Additional file 3: Table S3). Around half of these proteins (390 of 772) belonged to the core genome while 382 were not conserved across all 20 strains.

Twenty-seven protein sequences had a single transmembrane domain and also contain LPXTG motifs (Additional file 3: Table S3). Twenty-six of these proteins belonged to the accessory proteome, including 20 that are part of different *spa* gene clusters. Only one gene with an LPXTG motif, DIP0443, was conserved. LPXTG proteins contain a cell wall anchor domain and often play important roles in surface adhesion [40, 41]. The difference in the number of these proteins might be responsible for the variation in the adhesive properties between different *C. diphtheriae* strains. Additional 140 proteins were detected with a single transmembrane domain that might potentially be the peripheral membrane proteins or have domains projecting through the cell envelope, as in the case of the HtaA, HtaB, ChtA and ChtC proteins involved in scavenging of iron from host haemoglobin-haptoglobin [42].

Five hundred fourteen proteins were detected with two or more transmembrane domains and are predicted integral membrane proteins including two YidC proteins (Additional file 3: Table S3). One of the YidC proteins (DIP2379) is conserved in all 20 strains whereas DIP2273 is missing in one strain, NCTC 3529 (Additional file 3: Table S3). YidC proteins are involved in insertion of integral membrane proteins into the membrane [43]. The virulence factor DIP0733 (67-72p hemagglutinin) is an example of a core membrane protein, with 7 predicted transmembrane domains and a large C-terminal domain that appears to project through the cell envelope allowing interaction with host cell components promoting adherence and invasion [44–46]. Likewise, the previously characterised sialidase NanH [47] is a putative C-terminally membrane anchored protein. Although part of the core proteome, this gene is noted to show considerable allelic variation across the *C. diphtheriae* strains (for example, the originally described NanH sequence is only 76 % amino acid identical to DIP0543).

Fifty-three proteins with transmembrane domains were also predicted to have signal peptides (Additional file 3: Table S3). Some of these proteins were annotated to be ABC transporters, membrane anchored proteins, putative secreted proteins and 29 were hypothetical proteins. 21 of the 53 proteins were assigned to various COG categories involved in different cellular activities while no category was assigned to the remaining 32 proteins (Fig. 3c).

### The predicted secreted proteome of *C. diphtheriae*

Of the entire proteome, 559 proteins (14 %) are predicted to be secreted via Sec-dependent, Tat or type VII secretion pathways (Additional file 3: Table S3). Of these, 61 secreted proteins are predicted to be lipoproteins and 37 of these are conserved in all *C. diphtheriae* strains (Additional file 3: Table S3). Predicted lipoproteins constitute 2.3 % (37/1625) of the core proteome. As in other Actinobacteria [48–50], the major functional class identified are substrate binding proteins of ABC transport systems. In contrast to soil dwelling bacteria, where substrate binding proteins for sugars predominate, the predominant functional classes of substrate binding proteins in *C. diphtheriae* belong to families for amino acid/peptide uptake (e.g., PF00496) and for Fe^3+^/siderophore uptake (e.g., PF01497), presumably reflecting different nutrient availability in environmental versus host niches. The Fe^3+^/siderophore substrate binding proteins include the previously identified HmuT and CiuA lipoproteins [42, 51, 52]. Other functional categories included several predicted lipoproteins with predicted functions related to cell wall homeostasis (e.g., penicillin-binding protein DIP1497) or roles in redox processes, including the thioredoxin-like protein DIP0411 [53] which belongs to a locus (DIP0411-0414) involved in maturation of heme-containing cytochromes. The cytochrome C oxidase component CtaC (DIP1629) is also a putative lipoprotein, with two additional transmembrane domains (Additional file 3: Table S3), as in *Corynebacterium glutamicum* [54]. As in other Actinobacteria, the core lipoproteome includes the LpqN lipoprotein that likely represents an additional component of the MtrAB two-component sensing system [55]. Finally, 13 out of 37 (29 %) predicted lipoproteins in the core lipoproteome are hypothetical proteins of unknown function.

Eighty-three predicted secreted proteins were identified with a SpI type signal peptide (Fig. 3d; Additional file 3: Table S3). The BLAST searches of these protein sequences in the COG database returned no hits for 52 of these proteins (62.7 %) and 7 (8.4 %) were assigned to have general or no predicted functions. The remaining secreted proteins were assigned to different COG categories involved in a variety of cellular activities. Most notable of these is the phage-encoded diphtheria toxin (DIP0222), which is part of the accessory proteome (being absent from non-toxigenic strains) and plays a central role in the pathogenesis of diphtheria [22, 23]. Of 34 secreted proteins identified in the core proteome, two major functional categories were observed: degradative enzymes (mainly proteolytic e.g., DIP0836 and DIP1509, PF01551 peptidase M23 family members) and 14 proteins likely to be active in cell envelope homeostasis. These latter include DIP2191, a putative lipase likely involved in regulating cell envelope lipid composition [56, 57]. DIP2191 is located in the putative cell wall core biosynthetic locus, which also includes the secreted proteins DIP2193 and DIP2194 which are predicted to be mycolyltransferases involved in cell envelope assembly [58]. Notably, the 14 core proteins with signal peptides that are predicted to be involved in cell envelope homeostasis may not be fully secreted as translocation across the plasma membrane most likely results in a location in the ‘pseudoperiplasm’ created by the mycolate outer membrane [58]. These proteins also include two other proteins previously linked to *C. diphtheriae* virulence, DIP1281 and DIP1621 [59, 60], which are putative peptidoglycan endopeptidases (NlpC/P60 domain) proteins. Intriguingly, when compared to mycobacterial RipA and RipB [61], the catalytic site cysteine and histidine residues are conserved in both DIP1281 and DIP1621, whereas the catalytic site glutamate is only conserved in DIP1281. In DIP1621, the corresponding residue is a histidine (data not shown), as is typical in prototypical NlpC/P60 domain proteins, although it is notable that at Glu to His mutation inactivated mycobacterial RipA [61]. This sequence change (which may reflect an altered substrate specificity) and the presence of different additional domains in DIP1281 and DIP1621 point towards distinctive roles in cell wall remodelling in *C. diphtheriae*.

Tat signal peptides were identified in six proteins including four with a lipobox (Additional file 3: Table S3). Two of these, DIP0067 and DIP1389, are likely involved in redox processes. DIP0067 is a putative multi-copper oxidase homologous to the *M. tuberculosis* Rv0846c Tat lipoprotein linked to copper resistance [62] and its function likely involves the copper binding lipoprotein DIP0066. DIP1389 is a putative Dyp-family peroxidase and its function likely relies on interaction with the DIP1390 lipoprotein, which is a putative copper-binding protein. Both of the non-lipoprotein Tat substrates contain domains of unknown function, of which DIP0793 contains a PF08924 (BacA-like) putative peptidoglycan hydrolase domain.

In addition to the Sec and Tat translocase, a single type VII secretion system was identified in the *C. diphtheriae* core proteome, consistent with previous analyses [63]. The core components of the system (EccB, EccC, EccD and a mycP protease) were present in a locus (DIP0552-DIP0559) which also contains a type VII- secretion associated protein (Rv3446c family member) and two type VII secretion substrates (WXG100 family protein, DIP0558 and DIP0559). These latter were the only WXG100 family (PF06013) members detected in the *C. diphtheriae* genomes, suggesting these are the principal substrates for this specialised secretion system, which is known to influence virulence in related bacteria [63].

A large proportion of the putative secreted proteins (407/549 proteins) was predicted to be non-classical secreted proteins without any signal peptide, although only 49 of them are conserved in all 20 *C. diphtheriae* genomes (Additional file 3: Table S3). Twenty-eight of these proteins are assigned to the COG category involved in DNA replication and metabolism and 14 to the one involved in translation, ribosomal structure and biogenesis (Fig. 3d). A small fraction (1–7 proteins) are assigned to diverse COG categories including defence mechanisms but the majority (79.6 %) of these proteins could not be assigned to any functional category including those with general function prediction or no known functions (Fig. 3e; Additional file 3: Table S3). A small protein (82–108 aa in size) annotated as preprotein translocase subunit in seven strains (C7*β*, HC04, PW8, ISS4060, NCTC5011, NCTC3529 and Aberdeen) was predicted to be a non-classical secreted protein (Additional file 3: Table S3). A search in the UniProt database revealed this protein to be YidC. Some of non-classical secreted proteins have been annotated to facilitate cellular activities including those associated with mobile genetic elements (transposases or phage associated; Additional file 3: Table S3) that may be moon-lighting [36]. However, these predictions are based on the search in a protein database which include proteins known to be secreted via Sec-independent pathways in other bacteria [36] and may not necessarily be relevant to *C. diphtheriae*.

### Cytoplasmic proteins

None of the prediction programs assigned any location (secreted or membrane) to 2,485 proteins and these were considered to be cytoplasmic proteins. As would be expected, these were predicted to be involved in a diverse range of cellular functions with 44 % of them classified as hypothetical proteins (Additional file 3: Table S3). Of these, 227 (9 %) proteins were involved in DNA replication, recombination and repair, 120 (5 %) in translation, ribosomal structure or biogenesis, 96 (4 %) in transcription, 92 (4 %) in amino acid transport and metabolism, 84 (3 %) in coenzyme transport and metabolism and 65 (~3 %) in defence mechanisms (Fig. 3e; Additional file 3: Table S3). Only 3–55 (≤2 %) were involved in other cellular functions including energy production and conversion, cell cycle control, cell division or chromosome partitioning, post-translational modification, protein turnover and chaperones, cell wall or membrane biogenesis, signal transduction, intracellular trafficking, and metabolism and transport of secondary metabolites, nucleotides, carbohydrates, lipids and inorganic ions. An additional 256 (10 %) cytoplasmic proteins were potentially involved in multiple functions and were assigned to more than one functional category (Fig. 3e; Additional file 3: Table S3). However, these functions are assigned based on similarities of proteins in the COG database and may not be precise. The BLAST search did not reveal any functional categories for rest of the proteins including those with general or unknown functions (COG categories R and S).

### Phylogenetic relatedness among *C. diphtheriae* strains

**Fig. 4:**
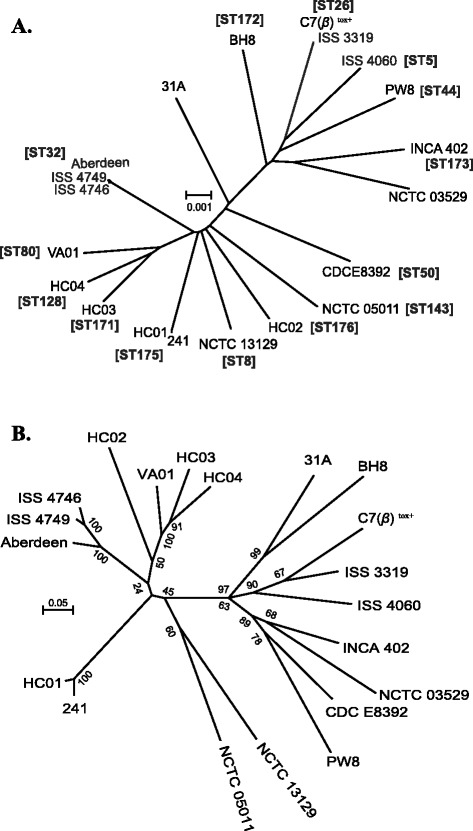
**a** A neighbour-joining phylogenetic tree from the variation in the core genome (1625 CDS) of 20 *C. diphtheriae* strains. The scale bar represents the number of substitutions per site. ST designations of the strains are mapped on the tree in the parentheses. **b** A maximum-likelihood tree from the binary data of the presence or absence of genes in the accessory genome. The scale bar with a distance of 0.05 represents the difference of 118.2 genes

A phylogenetic tree derived from the conserved core genome revealed that ISS 4746 and ISS 4749 are closely related to the strain ‘Aberdeen’ that also belongs to ST32 (Fig. 4a). ISS 3319 was closely related to strain C7(*β*)^tox+^ (ST26) whilst strains HC01 and 241 also have an almost identical core genome (ST175; Fig. 4a). The other strains, including ISS 4060, were phylogenetically distinct from each other, that is in agreement with our previous studies [24, 25]. Similar groupings were obtained when a phylogenetic tree was generated from the amino acid sequences using PhyloPhlAn (Additional file 4: Figure S1) [64]. PhyloPhlAn generates highly robust phylogenetic trees from a concatenated alignment of a computationally selected subset of amino-acid sequences from 400 most conserved universal proteins following a maximum likelihood maximization approach using RAxML [64, 65].

A maximum likelihood tree from the binary data based on the presence or absence of the genes in the accessory genome revealed that most of the *C. diphtheriae* strains were distantly related to each other (Fig. 4b) which is consistent with the phylogenetic relatedness from the core genome and universal protein sequences (Fig. 4a). The closely related strains ISS 4746, ISS 4749 and ‘Aberdeen’, and HC01 and 241 were marginally seperated from each other, indicating minor variations in the gene content of the strains within each ST or clonal group (Fig. 4b). However, C7(*β*)^tox+^ and ISS 3319 were observed to be relatively distant to each other, suggesting that the accessory genome can be quite different between individual strains within a ST. Most of the variaton in the accessory genome of *C. diphtheriae* is contributed by the genomic islands that are horizontally acquired [18].

## Discussion

### *C. diphtheriae* pan-genome and cellular locations of the encoded proteins

The pan-genome of *C. diphtheriae* was calculated to have 3,989 genes including 1,625 core genes and 2,364 accessory genes (Additional file 3: Table S3). The size of the pan-genome has reduced in comparison to previous studies calculating 4,789 genes for 13 *C. diphtheriae* strains [18] and 4,918 genes for 17 genomes [25]. EDGAR uses a cut-off score ratio value (SRV) to identify orthologous genes that is estimated by a sliding window approach as previously described [18, 26]. We have used an amended approach to estimate the cut-off SRV to include fitting of a *β*-distribution to the lower 50 % of observed scores to model the SRV peak with random hits. 97 percentile of this peak was calculated and used as the cut-off. This new approach reduces the chances of overestimation of the pan-genome and has been tested on a variety of datasets (J. Blom, unpublished data). Those genes with partial sequences due to the draft status of seven genomes were excluded from the calculation, which may have also partly reduced the number of genes in the pan-genome.

One thousand nine hundred fifty-eight genes (49 %) of the pan-genome were predicted to encode hypothetical proteins of which 569 genes (29.1 %) belong to the core genome and 1,389 genes (70.9 %) to the accessory genome (Additional file 3: Table S3). These results are consistent with our previous observation that the majority of variation in the gene content between *C. diphtheriae* strains is in the genes encoding hypothetical proteins [25]. Uncharacterised or hypothetical proteins impose major barriers in understanding the biology of an organism. Our analyses using advanced bioinformatics tools predicted 62 % of the whole proteome to be cytoplasmic, 14 % to be secretory and 19 % as transmembrane proteins (Additional file 3: Table S3; Fig. 3a–b). These results are in agreement with a previous study showing 15–30 % of the proteins in prokaryotic cells contain transmembrane helices while 15–25 % are secreted [66].

More than 61 % of the accessory genome (1,448/2,364 genes) encodes cytoplasmic proteins indicating a potential variation in the lifestyle of different strains. This finding is consistent with our previous observation showing the absence of several genes involved in carbohydrate metabolism in strain NCTC 5011 of lipophilic biovar intermedius that needs lipids for optimal growth [25].

Bacterial secreted proteins are translocated via a number of mechanisms including Sec, Tat or type VII secretion pathways [67, 68]. In addition, some non-classical secreted proteins that lack a signal peptide have also been found to be present in extracellular environment that may be secreted via non-classical pathways or ‘piggy-back’ out on proteins with classical signal peptides [36, 69–73]. Secreted and transmembrane proteins exhibit great differences between *C. diphtheriae* strains and are also important in determining host-pathogen interactions and pathogenesis [17, 20, 27, 28, 59, 68, 74]. These data are consistent with this hypothesis; however, the BLAST searches in the COG database could not assign any functional category to 40-80 % of these proteins and scored some of them as general function prediction proteins or proteins with unknown functions (COG codes R and S, respectively).

The phylogenetic analysis based on the presence or absence of the genes in the accessory genome distinctly separated most of the strains indicating the presence of strain-specific genes (Fig. 4b). Indeed, 11 of 82 genes were strain-specific and were not shared between *C. diphtheriae* strains (Additional file 3: Table S3). Trost et al. reported the existence of 57 genomic islands in *C. diphtheriae*, with varying distributions, some being strain specific to eight that were shared by all the strains [18]. These islands contribute to the variation in the gene composition among *C. diphtheriae* strains, which is consistent with our findings.

### Reliability of the predicted proteome

The genome wide categorisation of proteins as transmembrane, secreted and cellular proteins is based on *in silico* analyses using an array of different prediction programs and the reliability of these assignments is an important question. The predicted protein assignments in this study are quite reliable; first, all the programs have been largely used by the community and tested on a variety of datasets and secondly, we followed a stringent prediction strategy by using at least two different programs to predict proteins with transmembrane domains [34, 35] and signal peptides [34, 75], including lipoproteins [76, 77] and those secreted by the Tat pathway [78, 79]. The categories were assigned based on a consensus between these programs and the proteins were treated as ambiguous in case of a lack of agreement between different predictors. Some of the ambiguities were resolved by the manual analysis of the protein sequences. However, only one program was available to identify non-classical secreted proteins but these predictions are based on the homology with the proteins experimentally shown to be secreted by different groups [36].

A proteomic analysis of *C. diphtheriae* strain C7_s_(−)^tox-^ identified 50 secreted, 11 membrane-associated and 24 proteins detected both extracellularly and in the membrane fraction [80]. Many of these proteins are encoded by the pathogenicity islands and may be involved in host-pathogen interaction and virulence. Only 14 of the 50 extracellular proteins were assigned to be secreted, 21 were cytoplasmic, 11 with transmembrane domains and four were ambiguous, based on our prediction (Additional file 3: Table S3). 16/24 proteins reported both in the extracellular environment and the cell surface were detected to have signal peptides and one of them also had a single transmembrane domain. The remaining 8/24 proteins were predicted to be cytoplasmic. Similarly, transmembrane domains were only detected in 3/11 cell surface proteins (Additional file 3: Table S3). As suggested by Hansmeier et al., most of these discrepancies might be due to cross-contamination of protein fractions during the sample processing [80] while some might belong to putative non-classical secretome of *C. diphtheriae* that is not available in the database used by the program SecretomeP 2.0 [36].

The lipoprotein prediction in this study has also been reasonably consistent with the DOLOP database [81, 82]. 31 of the 42 lipoproteins in the DOLOP database for strain NCTC 13129 were correctly predicted by the approach applied in this study. A lipobox was also present in 3 Tat proteins but remaining 8 proteins were assigned to be secreted, transmembrane or cytoplasmic with no lipobox detected (Additional file 3: Table S3). This confirms that we have followed a robust strategy to assign proteins to different categories. The minor variations in the predictions might reflect the methodological variations followed by different programs to identify signal peptides.

### Proteomic variations within a *C. diphtheriae* clone

Bacterial isolates that belong to the same ST are typically treated as a single bacterial clone [83, 84]. In our analyses, strains C7(*β*)^tox+^ and ISS 3319 were found to belong to ST26; strains ISS 4746, ISS 4749 and Aberdeen to belong to ST32; and strains HC01 and 241 to ST175 (Fig. 4a). 15–290 proteins were strain specific within a clone that were not shared with other members of the same ST (data not shown). These specific genes may have introduced minor variations in functional and virulence characteristics between different strains within each ST as the proportion of these genes was highly variable between cytoplasmic (51–71 %), secreted (9–27 %) and transmembrane proteins (4–22 %), depending on the strain. Non-classical secreted proteins constituted most of the variations in the predicted secreted proteins; however, some of the secreted proteins were clearly involved in virulence, for example, C7(*β*)^tox+^ carried the *tox* gene that were absent in ISS 3319 within ST26 (Additional file 3: Table S3). This reflects in vitro lysogenization of the non-toxigenic C7(−) strain by corynephage *β* that converted it into the toxigenic C7(*β*)^tox+^ strain [85, 86]. Similarly, a YidC like protein was present in strain ‘Aberdeen’ that was absent in the other strains within ST32.

The organisation and functional structures of *spa* operons were quite similar within each ST with minor exceptions (Fig. 2) [18]. ISS 3319 and C7(*β*)^tox+^ have identical SpaD and SpaH gene clusters except for the presence of an additional gene encoding a hypothetical protein (AL06_01315) in the SpaD cluster of ISS 3319 (Fig. 2) [18]. All three Spa clusters (SpaA, SpaD and SpaH) were identical between HC01 and 241 [18] as well as ISS 4746 and ISS 4749 (Fig. 2). However, the *spaB* gene of the SpaA cluster is a pseudogene in ISS 4746 whereas it is intact in ISS 4749 (Fig. 2). It is possible that these minor variations due to gain or loss of the gene functions as well as some of the uncharacterised strain-specific genes may contribute to variations in strain fitness or virulence between individuals within an ST.

### Proteomic variations associated with the degree of virulence

The main objective of this study was to identify genes that could potentially be associated with the variation in the invasiveness between non-toxigenic *C. diphtheriae* strains ISS 3319, ISS 4060, ISS 4746 and ISS 4749. These strains are phylogenetically distinct and carry genes involved in a variety of cellular and metabolic activities including transcription, DNA replication and repair as well as defence mechanisms that are not common among them (Fig. 4a–b, Additional file 3: Table S3). ISS 4746 and ISS 4749 were the exceptions in that they are closely related and share most of their genomes with each other (Fig. 1, Table 2, Additional file 1: Table S1 and Additional file 3: Table S3).

*spa* operons encode adhesive surface pili that play important role in interaction with the host cells [27, 28]. In agreement with previous observations, the numbers and organisation of *spa* operons were found to be variable between these strains (Fig. 2) [18]. SpaD and SpaH gene clusters are present in all four strains but an additional SpaA gene cluster is present in ISS 4746 and ISS 4749 (Fig. 2). SpaA type pili have been found to be involved in adhesion to pharyngeal epithelial cells while SpaD and SpaH type pili are required for interaction with laryngeal and lung epithelial cells [87, 88]. Both the strains with three Spa gene clusters showed higher adhesion to the pharyngeal D562 cell lines [17]. The numbers of surface pili were found to be higher in ISS 4749 compared to the other strains [17]; however, the gene *spaF* which encodes a surface anchored fimbrial subunit in SpaD type pili, is a pseudogene in this strain and it is possible that the observed pili are the result of the expression of only the SpaA and SpaH clusters. An additional gene, *spaB* of the SpaA gene cluster, is a pseudogene in ISS 4746 (Fig. 2). The *spaB* gene encodes the pilus base subunit that works as a molecular switch to terminate the polymerization process and anchors the pilus polymer to the bacterial cell wall [27, 28, 88]. The inactivation of this gene results in the extracellular secretion of the pilus [88]. Therefore, an expression of only SpaH cluster may be associated with the lower number of surface pili in this strain [17]. Only the SpaD gene cluster appears to be fully functional in ISS 3319 which also correlates with a relatively low expression of surface pili in comparison to ISS 4749 [17]. Surprisingly, despite the presence of all the genes in the SpaH operon (Fig. 2), no pili were observed on the surface of ISS 4060 [17]. However, the efficiency with which this strain could adhere to the pharyngeal cell line D562, cervix carcinoma cell line HeLa and laryngeal cell line HEp-2 was comparable to that of ISS 3310 [13, 17, 89], indicating that the expression of SpaH pili is regulated by other factors in ISS 4060 or that alternative adhesins are significant in this strain, for example, lipoarabinomannan-like lipoglycan (CdiLAM) [90] and non-fimbrial surface proteins [44, 45].

Strains ISS 4746 and ISS 4749 were more invasive causing articular lesions in 50–60 % of the infected mice in comparison to only 25 % by ISS 3319 [15]. The former two strains also induced much higher local levels of interleukin (IL)-6 and IL-1 *β* production than the latter, which induced higher secretion of interferon-γ [15]. Relatively high number of the secreted and membrane associated proteins (including LPXTG proteins) were detected in ISS 4746 and ISS 4749 compared to ISS 3319 (Additional file 3: Table S3). This variation in the secreted and membrane proteins might be responsible for the variation in the invasiveness of these strains. Most of these proteins are uncharacterised and a molecular characterization of these proteins might improve our understanding of the mechanisms of invasion by *C. diphtheriae*.

## Conclusions

In conclusion, non-toxigenic *C. diphtheriae* strains vary in their gene content including the presence or absence of the substrate binding proteins of ABC transporters of different functional categories and cytoplasmic proteins that are involved in various metabolic activities, suggesting a potential variation in nutritional requirements or ability to use different sources of energy between these strains. The variation in the proteins with transmembrane domains, including those involved in the pili synthesis and polymerization appears to correlate with the adherence properties of strains with different epithelial cells, potentially providing them with an advantage when colonizing different tissues/organs in the host. The differences in the secreted proteins might be responsible for the variation in the degree of pathogenesis between different non-toxigenic *C. diphtheriae* strains.

## Methods

### Bacterial strains and genome sequencing

Four clinical non-toxigenic *C. diphtheriae* strains ISS 3319, ISS 4060, ISS 4746 and ISS 4749 were isolated in Italy between 1997 and 2001 (Additional file 2: Table S2). These strains have been isolated and characterised using molecular and immunological approaches in previous studies [4, 15, 17]. No patient material or information is analysed in this study and therefore no further ethical approval was needed to use these strains.

The strains were grown on Brain-Heart Infusion agar overnight at 37 °C. Five ml Brain-Heart Infusion broth was inoculated with a single colony and incubated for 16 h at 37 °C in a shaking incubator. The genomic DNAs were extracted and sequenced on a Roche GS Junior instrument, as previously described [25, 31, 32]. The resulting reads were assembled using GS *de novo* assembler.

### Genomic analyses

The draft genomes were submitted to the GenBank for annotation by the NCBI Prokaryotic Genome Annotation Pipeline [91]. The gene contents of the sequenced strains were compared using EDGAR [26]. The calculation of the core and accessory genomes including publicly available genomes (Additional file 2: Table S2) were performed and a phylogenetic tree from the alignment of 558,658 amino acids from the core proteins was also generated using EDGAR [26]. The protein functional categories were obtained by protein BLAST searches to the Clusters of Orthologous Group (COG) database using an *E*-value cut-off of 0.001 and composition-corrected scoring approach [92, 93]. Multilocus sequence type (MLST) profiles from all the genomes were extracted using MLST 1.7 [94]. A phylogenetic tree from the universal genomic proteins was generated using PhyloPhlAn [64]. A maximum-likelihood tree was generated using RAxML [65] with 100 bootstrap replicates from the binary data based on the presence or absence of the genes (2,364 genes) in the accessory genome.

### Proteome prediction

Signal peptides were detected in the protein sequences using SignalP 4.1 with default settings for Gram-positive bacteria [75] and Phobius webserver [34]. Phobius was also used to detect transmembrane domains along with TMHMM 2.0 [35]. Lipoproteins were detected using LipoP 1.0 [76] and PRED_LIPO [77]. TatP 1.0 [78] and TATFIND 1.4 [79] were used for detecting Tat signal peptides. Non-classical secreted proteins were predicted using SecretomeP 2.0 [36].

The proteins with signal peptides detected both by SignalP v4.1 and Phobius were scored as secreted proteins and proteins with transmembrane domains detected by Phobius and TMHMM 2.0 were designated as membrane associated proteins. The secreted proteins with a lipobox detected both by LipoP 1.0 and PRED_LIPO programs were assigned to be lipoproteins. The proteins where only one of these programmes detected a lipobox were manually inspected, as previously described [95]. Secreted proteins were scored as Tat-secreted when both TatP 1.0 and TATFIND 1.4 identified a twin-arginine signal peptide in the sequence. CW-PRED [96] was used for identifying proteins with LPXTG domains that were manually inspected for the presence of a signal peptide and a single transmembrane domain. The proteins with a signal peptide and transmembrane domains were separated as membrane-bound secreted proteins. Some proteins where a signal peptide or a transmembrane domain was detected only by one of the programs were treated as ambiguous. Proteins only identified by SecretomeP 2.0 but not any other programs were scored as non-classical secretory proteins and rest of the proteins with no transmembrane domain or signal peptide were treated as cytoplasmic proteins (Additional file 3: Table S3).

## Availability of supporting data

The Whole Genome Shotgun projects for *C. diphtheriae* strains ISS 3319, ISS 4060, ISS 4746 and ISS 4749 have been deposited at DDBJ/EMBL/GenBank (National Center for Biotechnology Information, GenBank Database: http://www.ncbi.nlm.nih.gov/nuccore/) under the accession numbers JAQO00000000, JAQN00000000, JAQP00000000 and JAQQ00000000, respectively and are publicly available. The versions described in this paper are JAQO01000000, JAQN01000000, JAQP01000000 and JAQQ01000000, respectively.

## Additional files

Additional file 1: Table S1. A list of strains specific genes in *C. diphtheriae* strains ISS 3319, ISS 4060, ISS 4746 and ISS 4749. (PDF 162 kb) Additional file 2: Table S2. Details of *C. diphtheriae* strains analysed in this study. (PDF 54 kb) Additional file 3: Table S3. A list of entire predicted proteome for *C. diphtheriae* using an array of computational programs. (XLSX 1116 kb) Additional file 4: Figure S1. A phylogenetic tree from a subset of amino acids from 400 universally conserved protein sequences using PhyloPhlAn. (PDF 151 kb)

## Abbreviations

IL: Interleukin
ST: Sequence type
MLST: Multilocus sequence type
Tat: Twin-Arginine Translocation
COG: Clusters of Orthologous Genes
BLAST: Basic Local Alignment Search Tool
SRV: Score ratio value
