# Supplementary material for: Adherence and invasive properties of Corynebacterium diphtheriae strains correlates with the predicted membrane-associated and secreted proteome
Source: BMC Genomics. 2015 Oct 9;16:765. doi: 10.1186/s12864-015-1980-8 (PMC4600297; doi:10.1186/s12864-015-1980-8)
Supplement: Additional file 1: Table S1. — A list of strains specific genes in C. diphtheriae strains ISS 3319, ISS 4060, ISS 4746 and ISS 4749. (PDF 162 kb) [file 12864_2015_1980_MOESM1_ESM.pdf]

Table S1. List of strains specific genes in ISS 3319, ISS 4060, ISS 4746 and ISS 4749

Strain	Locus	Function	COG Code
ISS 3319	AL06_00195	type I restriction endonuclease subunit S	V
ISS 3319	AL06_00225	hypothetical protein	
ISS 3319	AL06_00630	hypothetical protein	
ISS 3319	AL06_00665	transposase	
ISS 3319	AL06_00670	hypothetical protein	
ISS 3319	AL06_00970	hypothetical protein	K
ISS 3319	AL06_00975	hypothetical protein	
ISS 3319	AL06_01315	hypothetical protein	
ISS 3319	AL06_01480	hypothetical protein	
ISS 3319	AL06_01490	hypothetical protein	
ISS 3319	AL06_01865	hypothetical protein	
ISS 3319	AL06_01890	hypothetical protein	
ISS 3319	AL06_02695	transposase	H
ISS 3319	AL06_02700	hypothetical protein	
ISS 3319	AL06_02705	hypothetical protein	
ISS 3319	AL06_02715	transposase	
ISS 3319	AL06_02720	hypothetical protein	
ISS 3319	AL06_02730	cell filamentation protein Fic	D, S
ISS 3319	AL06_02735	hypothetical protein	
ISS 3319	AL06_02740	hypothetical protein	
ISS 3319	AL06_02745	DNA methyltransferase	V
ISS 3319	AL06_02750	hypothetical protein	
ISS 3319	AL06_02755	hypothetical protein	
ISS 3319	AL06_02760	ATP-dependent helicase	
ISS 3319	AL06_02815	hypothetical protein	
ISS 3319	AL06_03145	hypothetical protein	
ISS 3319	AL06_03165	hypothetical protein	
ISS 3319	AL06_03760	hypothetical protein	
ISS 3319	AL06_03845	hypothetical protein	
ISS 3319	AL06_04085	hypothetical protein	
ISS 3319	AL06_04565	transposase	
ISS 3319	AL06_04760	ABC transporter ATP-binding protein	P
ISS 3319	AL06_04770	hypothetical protein	
ISS 3319	AL06_05150	hypothetical protein	
ISS 3319	AL06_05525	hypothetical protein	T
ISS 3319	AL06_05530	hypothetical protein	T
ISS 3319	AL06_05590	hypothetical protein	
ISS 3319	AL06_07200	hypothetical protein	
ISS 3319	AL06_08225	hypothetical protein	
ISS 3319	AL06_08575	surface-anchored membrane protein	
ISS 3319	AL06_08700	hypothetical protein	
ISS 3319	AL06_08945	hypothetical protein	
ISS 3319	AL06_08970	hypothetical protein	E
ISS 3319	AL06_09065	hypothetical protein	
ISS 3319	AL06_09075	hypothetical protein	
ISS 3319	AL06_09410	hypothetical protein	
ISS 3319	AL06_09415	hypothetical protein	

Strain	Locus	Function	COG Code
ISS 3319	AL06_09420	cadmium transporter	P
ISS 3319	AL06_09425	ArsR family transcriptional regulator	K
ISS 3319	AL06_09430	hypothetical protein	
ISS 3319	AL06_09435	hypothetical protein	
ISS 3319	AL06_09445	hypothetical protein	
ISS 3319	AL06_09450	hypothetical protein	
ISS 3319	AL06_09455	hypothetical protein	
ISS 3319	AL06_09830	hypothetical protein	
ISS 3319	AL06_09985	surface-anchored fimbrial subunit	
ISS 3319	AL06_09995	lipase	
ISS 3319	AL06_10060	hypothetical protein	
ISS 3319	AL06_10160	acetyltransferase	R
ISS 3319	AL06_10245	hypothetical protein	
ISS 3319	AL06_10250	hypothetical protein	
ISS 3319	AL06_10255	ABC transporter	P
ISS 3319	AL06_10260	hypothetical protein	
ISS 3319	AL06_10265	hypothetical protein	R
ISS 3319	AL06_10270	hypothetical protein	R
ISS 3319	AL06_10275	hypothetical protein	R
ISS 3319	AL06_10280	hypothetical protein	
ISS 3319	AL06_10290	transposase	L
ISS 3319	AL06_10410	hypothetical protein	
ISS 3319	AL06_10420	hypothetical protein	
ISS 3319	AL06_10455	hypothetical protein	O
ISS 3319	AL06_10515	hypothetical protein	
ISS 3319	AL06_10605	hypothetical protein	
ISS 3319	AL06_10745	hypothetical protein	
ISS 3319	AL06_10750	multidrug ABC transporter ATP-binding protein	V
ISS 3319	AL06_10755	multidrug ABC transporter ATP-binding protein	V
ISS 3319	AL06_10760	hypothetical protein	
ISS 3319	AL06_10765	hypothetical protein	
ISS 3319	AL06_10770	ABC transporter substrate-binding protein	E
ISS 3319	AL06_10775	ABC transporter permease	E, P
ISS 3319	AL06_10780	ABC transporter permease	E, P
ISS 3319	AL06_10885	hypothetical protein	
ISS 3319	AL06_10895	hypothetical protein	
ISS 3319	AL06_10995	DNA-binding protein	S
ISS 3319	AL06_11010	hypothetical protein	
ISS 3319	AL06_11015	hypothetical protein	
ISS 3319	AL06_11020	hypothetical protein	
ISS 3319	AL06_11025	toxin HicA	
ISS 3319	AL06_11030	transposase	T
ISS 3319	AL06_11040	membrane protein	
ISS 3319	AL06_11045	cupin	S
ISS 3319	AL06_11050	carboxymuconolactone decarboxylase	S
ISS 3319	AL06_11055	hypothetical protein	S
ISS 3319	AL06_11060	2,5-diketo-D-gluconic acid reductase	R
ISS 3319	AL06_11065	transcriptional regulator	K
ISS 3319	AL06_11075	hypothetical protein	

Strain	Locus	Function	COG Code
ISS 3319	AL06_11105	hypothetical protein	
ISS 3319	AL06_11215	hypothetical protein	R
ISS 3319	AL06_11220	quinolinate synthetase	H
ISS 3319	AL06_11225	L-aspartate oxidase	E
ISS 3319	AL06_11230	nicotinate-nucleotide pyrophosphorylase	H
ISS 3319	AL06_11305	TetR family transcriptional regulator	K
ISS 3319	AL06_11365	hypothetical protein	
ISS 4060	AL07_00105	preprotein translocase subunit	S
ISS 4060	AL07_00215	hypothetical protein	
ISS 4060	AL07_00410	mercuric reductase	C
ISS 4060	AL07_00415	redoxin	C, O
ISS 4060	AL07_00420	cytochrome C biogenesis protein	C, O
ISS 4060	AL07_00425	hypothetical protein	
ISS 4060	AL07_00430	MerR family transcriptional regulator	K
ISS 4060	AL07_00435	alkyl hydroperoxide reductase	
ISS 4060	AL07_00440	hypothetical protein	
ISS 4060	AL07_00450	cation transporter	P
ISS 4060	AL07_00455	cation transporter	P
ISS 4060	AL07_00460	BetI family transcriptional regulator	K
ISS 4060	AL07_00465	FAD-dependent pyridine nucleotide-disulfide oxidoreductase	G, P
ISS 4060	AL07_00470	ArsR family transcriptional regulator	K
ISS 4060	AL07_00475	arsenite transporter	P
ISS 4060	AL07_00480	arsenate reductase	T
ISS 4060	AL07_00485	hypothetical protein	
ISS 4060	AL07_00490	membrane protein	R
ISS 4060	AL07_00495	ArsR family transcriptional regulator	K
ISS 4060	AL07_00500	hypothetical protein	
ISS 4060	AL07_00505	hypothetical protein	
ISS 4060	AL07_00760	hypothetical protein	
ISS 4060	AL07_00775	transposase	
ISS 4060	AL07_00780	hypothetical protein	
ISS 4060	AL07_00790	hypothetical protein	
ISS 4060	AL07_00795	hypothetical protein	
ISS 4060	AL07_00800	hypothetical protein	
ISS 4060	AL07_01010	hypothetical protein	
ISS 4060	AL07_01025	hypothetical protein	
ISS 4060	AL07_01070	hypothetical protein	
ISS 4060	AL07_01100	hypothetical protein	
ISS 4060	AL07_01160	hypothetical protein	
ISS 4060	AL07_01235	hypothetical protein	L
ISS 4060	AL07_01285	transposase	
ISS 4060	AL07_01290	surface-anchored fimbrial subunit	M
ISS 4060	AL07_01565	hypothetical protein	
ISS 4060	AL07_01575	DEAD/DEAH box helicase	V
ISS 4060	AL07_01580	restriction endonuclease subunit S	V
ISS 4060	AL07_01585	toxin Fic	R
ISS 4060	AL07_01595	hypothetical protein	R
ISS 4060	AL07_01665	transposase	L
ISS 4060	AL07_02185	hypothetical protein	

Strain	Locus	Function	COG Code
ISS 4060	AL07_02190	hypothetical protein	
ISS 4060	AL07_02205	hypothetical protein	
ISS 4060	AL07_02210	transposase	
ISS 4060	AL07_02690	hypothetical protein	O
ISS 4060	AL07_02925	iron complex transporter substrate-binding protein	P
ISS 4060	AL07_03040	cell-surface hemin receptor	
ISS 4060	AL07_03045	transposase	L
ISS 4060	AL07_03050	hypothetical protein	
ISS 4060	AL07_03055	hypothetical protein	
ISS 4060	AL07_03080	hypothetical protein	
ISS 4060	AL07_03235	hypothetical protein	
ISS 4060	AL07_04310	calcium-binding protein	
ISS 4060	AL07_05235	hypothetical protein	L
ISS 4060	AL07_05580	hypothetical protein	
ISS 4060	AL07_06465	hypothetical protein	K, L
ISS 4060	AL07_06720	hypothetical protein	
ISS 4060	AL07_06900	hypothetical protein	
ISS 4060	AL07_07415	formate/nitrite transporter protein	P
ISS 4060	AL07_07930	transposase	L
ISS 4060	AL07_07935	DNA methylase	L
ISS 4060	AL07_07940	type III restriction enzyme	S, K, L
ISS 4060	AL07_07945	hypothetical protein	
ISS 4060	AL07_07950	hypothetical protein	
ISS 4060	AL07_08480	hypothetical protein	
ISS 4060	AL07_08770	hypothetical protein	
ISS 4060	AL07_09455	hypothetical protein	
ISS 4060	AL07_09520	hypothetical protein	
ISS 4060	AL07_09630	hypothetical protein	
ISS 4060	AL07_09645	hypothetical protein	
ISS 4060	AL07_09675	transposase ISRM3	
ISS 4060	AL07_09680	hypothetical protein	
ISS 4060	AL07_09700	transposase	
ISS 4060	AL07_09705	transposase	
ISS 4060	AL07_09735	hypothetical protein	
ISS 4060	AL07_09895	hypothetical protein	S
ISS 4060	AL07_09900	hypothetical protein	
ISS 4060	AL07_09905	hypothetical protein	
ISS 4060	AL07_09910	hypothetical protein	
ISS 4060	AL07_09915	hypothetical protein	
ISS 4060	AL07_10055	hypothetical protein	
ISS 4060	AL07_10095	hypothetical protein	
ISS 4060	AL07_10100	heat-shock protein	O
ISS 4060	AL07_10125	hypothetical protein	
ISS 4060	AL07_10255	hypothetical protein	
ISS 4060	AL07_10270	hypothetical protein	
ISS 4060	AL07_10280	transposase	
ISS 4060	AL07_10325	hypothetical protein	
ISS 4060	AL07_10520	hypothetical protein	
ISS 4060	AL07_10525	hypothetical protein	

Strain	Locus	Function	COG Code
ISS 4060	AL07_10530	collagen-binding protein	
ISS 4060	AL07_10535	hypothetical protein	
ISS 4060	AL07_10540	endo-beta-N-acetylglucosaminidase	
ISS 4060	AL07_10635	hypothetical protein	
ISS 4060	AL07_10640	hypothetical protein	L
ISS 4060	AL07_10645	hypothetical protein	
ISS 4060	AL07_10650	ABC transporter permease	P
ISS 4060	AL07_10655	ABC transporter ATP-binding protein	P
ISS 4060	AL07_10660	iron ABC transporter ATP-binding protein	V
ISS 4060	AL07_10665	ABC transporter	V
ISS 4060	AL07_10670	TetR family transcriptional regulator	K
ISS 4060	AL07_10675	hypothetical protein	
ISS 4060	AL07_10680	hypothetical protein	
ISS 4060	AL07_10685	hypothetical protein	
ISS 4060	AL07_10695	transposase	
ISS 4060	AL07_10700	hypothetical protein	
ISS 4060	AL07_10705	hypothetical protein	
ISS 4060	AL07_10710	plasmid maintenance system killer	R
ISS 4060	AL07_10715	transcriptional regulator	R
ISS 4060	AL07_10720	hypothetical protein	P
ISS 4060	AL07_10730	hypothetical protein	J, D
ISS 4060	AL07_10740	hypothetical protein	V
ISS 4060	AL07_10755	transposase	
ISS 4060	AL07_11170	hypothetical protein	V
ISS 4060	AL07_11185	hypothetical protein	
ISS 4060	AL07_11420	hypothetical protein	
ISS 4060	AL07_11430	transposase	L
ISS 4060	AL07_11435	hypothetical protein	S, F
ISS 4060	AL07_11450	hypothetical protein	
ISS 4060	AL07_11475	hypothetical protein	
ISS 4746	AL08_00005	hypothetical protein	L
ISS 4746	AL08_00010	hypothetical protein	
ISS 4746	AL08_02185	hypothetical protein	D
ISS 4746	AL08_02510	hypothetical protein	
ISS 4746	AL08_03065	MerR family transcriptional regulator	K
ISS 4746	AL08_03070	sulfate permease	P
ISS 4746	AL08_03075	hypothetical protein	
ISS 4746	AL08_03080	relaxase	
ISS 4746	AL08_03085	mobilization protein	
ISS 4746	AL08_03090	hypothetical protein	
ISS 4746	AL08_03095	hypothetical protein	
ISS 4746	AL08_03100	hypothetical protein	
ISS 4746	AL08_03105	hypothetical protein	
ISS 4746	AL08_03110	hypothetical protein	
ISS 4746	AL08_03115	hypothetical protein	
ISS 4746	AL08_03125	hypothetical protein	
ISS 4746	AL08_03190	hypothetical protein	
ISS 4746	AL08_03225	transposase	
ISS 4746	AL08_05900	hypothetical protein	

Strain	Locus	Function	COG Code
ISS 4746	AL08_05985	hypothetical protein	
ISS 4746	AL08_06995	hypothetical protein	
ISS 4746	AL08_07000	hypothetical protein	
ISS 4746	AL08_07010	hypothetical protein	
ISS 4746	AL08_07015	hypothetical protein	
ISS 4746	AL08_09610	hypothetical protein	M
ISS 4746	AL08_10210	transposase	L
ISS 4746	AL08_11400	hypothetical protein	
ISS 4746	AL08_11420	integrase	
ISS 4746	AL08_11445	transposase	L
ISS 4746	AL08_11460	hypothetical protein	
ISS 4749	AL09_00065	hypothetical protein	L
ISS 4749	AL09_00210	hypothetical protein	
ISS 4749	AL09_00395	transposase IS3525	L
ISS 4749	AL09_00850	hypothetical protein	
ISS 4749	AL09_02710	hypothetical protein	
ISS 4749	AL09_04220	RNA pseudouridine synthase	J
ISS 4749	AL09_05750	membrane protein	S
ISS 4749	AL09_09385	surface anchored protein	
ISS 4749	AL09_09400	hypothetical protein	
ISS 4749	AL09_09645	hydrolase	R
ISS 4749	AL09_10005	hypothetical protein	
ISS 4749	AL09_10140	hypothetical protein	M
ISS 4749	AL09_10520	hypothetical protein	G
ISS 4749	AL09_11300	hypothetical protein	L
ISS 4749	AL09_11335	hypothetical protein	
ISS 4746 - ISS 4749	AL08_00520, AL09_00725	hypothetical protein	
ISS 4746 - ISS 4749	AL08_00525, AL09_00730	type-2 fimbrial major subunit	
ISS 4746 - ISS 4749	AL08_00575, AL09_00005	HicB	R
ISS 4746 - ISS 4749	AL08_00580, AL09_00010	septum formation protein	E
ISS 4746 - ISS 4749	AL08_00585, AL09_00015	hypothetical protein	R
ISS 4746 - ISS 4749	AL08_00590, AL09_00020	hypothetical protein	
ISS 4746 - ISS 4749	AL08_00595, AL09_00025	hypothetical protein	
ISS 4746 - ISS 4749	AL08_00600, AL09_00030	hypothetical protein	
ISS 4746 - ISS 4749	AL08_00605, AL09_00035	abortive phage resistance protein	V
ISS 4746 - ISS 4749	AL08_00610, AL09_00040	hypothetical protein	V
ISS 4746 - ISS 4749	AL08_00615, AL09_00045	hypothetical protein	K, L
ISS 4746 - ISS 4749	AL08_00620, AL09_00050	DNA helicase	K, L
ISS 4746 - ISS 4749	AL08_00625, AL09_00055	hypothetical protein	
ISS 4746 - ISS 4749	AL08_01090, AL09_01220	hypothetical protein	
ISS 4746 - ISS 4749	AL08_01320, AL09_01450	hypothetical protein	
ISS 4746 - ISS 4749	AL08_01880, AL09_02010	hypothetical protein	
ISS 4746 - ISS 4749	AL08_02000, AL09_02130	HtaA domain protein	
ISS 4746 - ISS 4749	AL08_02005, AL09_02135	hypothetical protein	
ISS 4746 - ISS 4749	AL08_02115, AL09_02245	hypothetical protein	
ISS 4746 - ISS 4749	AL08_02520, AL09_02635	cell-surface hemin receptor	
ISS 4746 - ISS 4749	AL08_02685, AL09_02805	hypothetical protein	
ISS 4746 - ISS 4749	AL08_02700, AL09_02820	hypothetical protein	
ISS 4746 - ISS 4749	AL08_02955, AL09_03075	hypothetical protein	

Strain	Locus	Function	COG Code
ISS 4746 - ISS 4749	AL08_02965, AL09_03085	hypothetical protein	
ISS 4746 - ISS 4749	AL08_03380, AL09_03430	tyrosine recombinase XerC	
ISS 4746 - ISS 4749	AL08_03385, AL09_03470	hypothetical protein	
ISS 4746 - ISS 4749	AL08_03490, AL09_03575	hypothetical protein	
ISS 4746 - ISS 4749	AL08_03495, AL09_03580	hypothetical protein	
ISS 4746 - ISS 4749	AL08_03500, AL09_03585	hypothetical protein	
ISS 4746 - ISS 4749	AL08_04555, AL09_04640	hypothetical protein	
ISS 4746 - ISS 4749	AL08_04880, AL09_04965	TetR family transcriptional regulator	
ISS 4746 - ISS 4749	AL08_05405, AL09_05490	hypothetical protein	C
ISS 4746 - ISS 4749	AL08_05425, AL09_05510	hypothetical protein	
ISS 4746 - ISS 4749	AL08_05840, AL09_05925	hypothetical protein	
ISS 4746 - ISS 4749	AL08_06120, AL09_06200	transposase IS1249	L
ISS 4746 - ISS 4749	AL08_06125, AL09_06205	transposase	
ISS 4746 - ISS 4749	AL08_06200, AL09_06275	transposase	
ISS 4746 - ISS 4749	AL08_06245, AL09_06320	hypothetical protein	
ISS 4746 - ISS 4749	AL08_07005, AL09_07050	cell-surface hemin receptor	
ISS 4746 - ISS 4749	AL08_07590, AL09_07635	hypothetical protein	
ISS 4746 - ISS 4749	AL08_07595, AL09_07640	hypothetical protein	S
ISS 4746 - ISS 4749	AL08_07600, AL09_07645	hypothetical protein	
ISS 4746 - ISS 4749	AL08_07605, AL09_07650	transposase	L
ISS 4746 - ISS 4749	AL08_07610, AL09_07655	transposase	L
ISS 4746 - ISS 4749	AL08_07620, AL09_07665	hypothetical protein	
ISS 4746 - ISS 4749	AL08_07625, AL09_07670	hypothetical protein	
ISS 4746 - ISS 4749	AL08_07630, AL09_07675	hypothetical protein	
ISS 4746 - ISS 4749	AL08_07635, AL09_07680	hypothetical protein	M
ISS 4746 - ISS 4749	AL08_07640, AL09_07685	hypothetical protein	S
ISS 4746 - ISS 4749	AL08_07645, AL09_07690	hypothetical protein	
ISS 4746 - ISS 4749	AL08_07650, AL09_07695	hypothetical protein	
ISS 4746 - ISS 4749	AL08_08290, AL09_08335	hypothetical protein	
ISS 4746 - ISS 4749	AL08_08295, AL09_08340	hypothetical protein	
ISS 4746 - ISS 4749	AL08_08300, AL09_08345	3-oxoacyl-ACP synthase	
ISS 4746 - ISS 4749	AL08_08310, AL09_08355	acyl-CoA dehydrogenase	I
ISS 4746 - ISS 4749	AL08_08315, AL09_08360	MFS transporter permease	
ISS 4746 - ISS 4749	AL08_08320, AL09_08365	transposase	L
ISS 4746 - ISS 4749	AL08_08335, AL09_08380	transposase	
ISS 4746 - ISS 4749	AL08_08340, AL09_08385	transposase	
ISS 4746 - ISS 4749	AL08_08525, AL09_08570	hypothetical protein	
ISS 4746 - ISS 4749	AL08_08840, AL09_08885	hypothetical protein	
ISS 4746 - ISS 4749	AL08_08890, AL09_08935	phosphate ABC transporter ATP-binding protein	P
ISS 4746 - ISS 4749	AL08_08895, AL09_08940	phosphate ABC transporter permease	P
ISS 4746 - ISS 4749	AL08_08900, AL09_08945	phosphate ABC transporter permease	P
ISS 4746 - ISS 4749	AL08_08905, AL09_08950	ABC transporter substrate-binding protein	P
ISS 4746 - ISS 4749	AL08_09050, AL09_09095	hypothetical protein	
ISS 4746 - ISS 4749	AL08_09055, AL09_09100	stress-responsive transcriptional regulator	K, T
ISS 4746 - ISS 4749	AL08_09085, AL09_09130	hypothetical protein	
ISS 4746 - ISS 4749	AL08_09210, AL09_09255	hypothetical protein	
ISS 4746 - ISS 4749	AL08_09335, AL09_09380	surface-anchored membrane protein	
ISS 4746 - ISS 4749	AL08_09345, AL09_09390	fimbrial associated sortase	M
ISS 4746 - ISS 4749	AL08_09350, AL09_09395	surface-anchored fimbrial subunit	

Strain	Locus	Function	COG Code
ISS 4746 - ISS 4749	AL08_09360, AL09_09405	lipase	
ISS 4746 - ISS 4749	AL08_09370, AL09_09415	hypothetical protein	
ISS 4746 - ISS 4749	AL08_09375, AL09_09420	hypothetical protein	
ISS 4746 - ISS 4749	AL08_09380, AL09_09425	transposase	
ISS 4746 - ISS 4749	AL08_09390, AL09_09435	hypothetical protein	
ISS 4746 - ISS 4749	AL08_09395, AL09_09440	hypothetical protein	
ISS 4746 - ISS 4749	AL08_09400, AL09_09445	hypothetical protein	
ISS 4746 - ISS 4749	AL08_09405, AL09_09450	hypothetical protein	
ISS 4746 - ISS 4749	AL08_09415, AL09_09460	hypothetical protein	V
ISS 4746 - ISS 4749	AL08_09420, AL09_09465	12-oxophytodienoate reductase	J, C
ISS 4746 - ISS 4749	AL08_09425, AL09_09470	excinuclease ABC subunit A	L
ISS 4746 - ISS 4749	AL08_09430, AL09_09475	transposase	R
ISS 4746 - ISS 4749	AL08_09435, AL09_09480	peptidase M20	R
ISS 4746 - ISS 4749	AL08_09440, AL09_09485	DNA repair ATPase	D
ISS 4746 - ISS 4749	AL08_09445, AL09_09490	restriction endonuclease	V
ISS 4746 - ISS 4749	AL08_09450, AL09_09495	DNA methyltransferase	L
ISS 4746 - ISS 4749	AL08_09455, AL09_11325	transposase	
ISS 4746 - ISS 4749	AL08_09460, AL09_09500	hypothetical protein	
ISS 4746 - ISS 4749	AL08_09465, AL09_09505	hypothetical protein	
ISS 4746 - ISS 4749	AL08_09470, AL09_09510	hypothetical protein	
ISS 4746 - ISS 4749	AL08_09590, AL09_09630	surface anchored protein	
ISS 4746 - ISS 4749	AL08_09605, AL09_09650	surface-anchored fimbrial associated protein	
ISS 4746 - ISS 4749	AL08_09660, AL09_09700	hypothetical protein	
ISS 4746 - ISS 4749	AL08_09665, AL09_09705	hypothetical protein	
ISS 4746 - ISS 4749	AL08_09670, AL09_09710	transporter	E, G
ISS 4746 - ISS 4749	AL08_09675, AL09_09715	oxidoreductase	I
ISS 4746 - ISS 4749	AL08_09680, AL09_09720	hypothetical protein	S
ISS 4746 - ISS 4749	AL08_09685, AL09_09725	GntR family transcriptional regulator	K
ISS 4746 - ISS 4749	AL08_09690, AL09_09730	D-amino acid dehydrogenase subunit	R, E
ISS 4746 - ISS 4749	AL08_09695, AL09_09735	hypothetical protein	E
ISS 4746 - ISS 4749	AL08_09700, AL09_09740	hypothetical protein	
ISS 4746 - ISS 4749	AL08_09710, AL09_09750	hypothetical protein	
ISS 4746 - ISS 4749	AL08_09730, AL09_09755	hypothetical protein	
ISS 4746 - ISS 4749	AL08_09765, AL09_09790	hypothetical protein	
ISS 4746 - ISS 4749	AL08_09970, AL09_10015	hypothetical protein	
ISS 4746 - ISS 4749	AL08_10035, AL09_10080	hypothetical protein	
ISS 4746 - ISS 4749	AL08_10090, AL09_10135	hypothetical protein	
ISS 4746 - ISS 4749	AL08_10185, AL09_10225	spermidine/putrescine ABC transporter ATP-binding protein	H, P
ISS 4746 - ISS 4749	AL08_10190, AL09_10230	ABC transporter permease	P
ISS 4746 - ISS 4749	AL08_10195, AL09_10235	ABC transporter substrate-binding protein	P
ISS 4746 - ISS 4749	AL08_10205, AL09_10245	peptidylprolyl isomerase	R, K
ISS 4746 - ISS 4749	AL08_10215, AL09_10255	transposase	
ISS 4746 - ISS 4749	AL08_10220, AL09_10260	transposase	L
ISS 4746 - ISS 4749	AL08_10225, AL09_10265	transposase	L
ISS 4746 - ISS 4749	AL08_10230, AL09_10270	hypothetical protein	
ISS 4746 - ISS 4749	AL08_10235, AL09_10275	hypothetical protein	
ISS 4746 - ISS 4749	AL08_10240, AL09_10280	hypothetical protein	
ISS 4746 - ISS 4749	AL08_10245, AL09_10285	hypothetical protein	
ISS 4746 - ISS 4749	AL08_10250, AL09_10290	surface-anchored protein	

Strain	Locus	Function	COG Code
ISS 4746 - ISS 4749	AL08_10270, AL09_10310	collagen-binding protein	M
ISS 4746 - ISS 4749	AL08_10290, AL09_10335	methylase	
ISS 4746 - ISS 4749	AL08_10625, AL09_10675	DNA-binding protein	K
ISS 4746 - ISS 4749	AL08_10675, AL09_10720	DNA-binding protein	
ISS 4746 - ISS 4749	AL08_10690, AL09_10735	type I restriction endonuclease subunit S	V
ISS 4746 - ISS 4749	AL08_10770, AL09_10815	SWI/SNF complex subunit SMARCC2	
ISS 4746 - ISS 4749	AL08_11105, AL09_11155	hypothetical protein	G
ISS 4746 - ISS 4749	AL08_11135, AL09_11185	membrane protein	L
ISS 4746 - ISS 4749	AL08_11295, AL09_00165	hypothetical protein	
ISS 4746 - ISS 4749	AL08_11300, AL09_00170	hypothetical protein	
ISS 4746 - ISS 4749	AL08_11305, AL09_00175	hypothetical protein	
ISS 4746 - ISS 4749	AL08_11310, AL09_00180	hypothetical protein	
ISS 4746 - ISS 4749	AL08_11315, AL09_00185	hypothetical protein	
ISS 4746 - ISS 4749	AL08_11320, AL09_00190	hypothetical protein	
ISS 4746 - ISS 4749	AL08_11325, AL09_00195	hypothetical protein	
ISS 4746 - ISS 4749	AL08_11335, AL09_00205	hypothetical protein	
ISS 4746 - ISS 4749	AL08_11340, AL09_11265	hypothetical protein	
ISS 4746 - ISS 4749	AL08_11345, AL09_11260	hypothetical protein	
ISS 4746 - ISS 4749	AL08_11350, AL09_11255	type II restriction endonuclease	
ISS 4746 - ISS 4749	AL08_11355, AL09_11250	modification methylase SalI	V
ISS 4746 - ISS 4749	AL08_11375, AL09_03435	hypothetical protein	
ISS 4746 - ISS 4749	AL08_11380, AL09_03440	hypothetical protein	
ISS 4746 - ISS 4749	AL08_11385, AL09_03445	integrase	
ISS 4746 - ISS 4749	AL08_11390, AL09_03450	hypothetical protein	
ISS 4746 - ISS 4749	AL08_11395, AL09_03455	hypothetical protein	
ISS 4746 - ISS 4749	AL08_11410, AL09_03460	hypothetical protein	
ISS 4746 - ISS 4749	AL08_11415, AL09_03465	hypothetical protein	
ISS 4746 - ISS 4749	AL08_11435, AL09_11270	hypothetical protein	L

Details of COG codes:
CELLULAR PROCESSES AND SIGNALING
[D] Cell cycle control, cell division, chromosome partitioning
[M] Cell wall/membrane/envelope biogenesis
[N] Cell motility
[O] Post-translational modification, protein turnover, and chaperones
[T] Signal transduction mechanisms
[U] Intracellular trafficking, secretion, and vesicular transport
[V] Defense mechanisms
[W] Extracellular structures
[Y] Nuclear structure
[Z] Cytoskeleton

INFORMATION STORAGE AND PROCESSING
[A] RNA processing and modification
[B] Chromatin structure and dynamics
[J] Translation, ribosomal structure and biogenesis
[K] Transcription
[L] Replication, recombination and repair

METABOLISM
[C] Energy production and conversion
[E] Amino acid transport and metabolism
[F] Nucleotide transport and metabolism
[G] Carbohydrate transport and metabolism
[H] Coenzyme transport and metabolism
[I] Lipid transport and metabolism
[P] Inorganic ion transport and metabolism
[Q] Secondary metabolites biosynthesis, transport, and catabolism

POORLY CHARACTERIZED
[R] General function prediction only
[S] Function unknown
