# Supplementary material for: Adherence and invasive properties of Corynebacterium diphtheriae strains correlates with the predicted membrane-associated and secreted proteome
Source: BMC Genomics. 2015 Oct 9;16:765. doi: 10.1186/s12864-015-1980-8 (PMC4600297; doi:10.1186/s12864-015-1980-8)
Supplement: Additional file 2: Table S2. — Details of C. diphtheriae strains analysed in this study. (PDF 54 kb) [file 12864_2015_1980_MOESM2_ESM.pdf]

Table S2. Details of *C. diphtheriae* strains

Strain	Biovar	Country	Year of isolation	GenBank accession no.	ST	tox gene	Source	Reference
PW8	mitis	USA	1896	CP003216	44	+	Diphtheria	[1]
NCTC 03529	mitis	UK	< 1932	AJGI00000000	-	+	Diphtheria	[2]
NCTC 05011	intermedius	UK	< 1932	AJVH01000000	143	+	Diphtheria	[3]
C7 (β)$^{tox+}$	mitis		1954	CP003210	26	+	Lab strain	[1]
31A	-	Brazil	1978	CP003206	-	+	Diphtheria	[1]
NCTC 13129	gravis	UK	1997	BX248353	8	+	Diphtheria	[4]
CDC E8392	mitis	USA		CP003211	50	+	Diphtheria	[1]
241	-	Brazil	1981	CP003207	175	-	Diphtheria	[1]
VA01	gravis	Brazil	1999	CP003217	80	-	Diphtheria	[1]
HC01	mitis	Brazil	1993	CP003212	175	-	Fatal endocarditis	[1]
HC02	mitis	Brazil	1999	CP003213	176	-	Endocarditis	[1]
HC03	mitis	Brazil	2000	CP003214	171	-	Endocarditis	[1]
HC04	gravis	Brazil	2003	CP003215	128	-	Fatal endocarditis	[1]
INCA 402	belfanti	Brazil	2000	CP003208	173	-	Pneumonia (cancer patient)	[1]
BH8	-	Brazil		CP003209	172	-	Antibiotic resistant	[1]
Aberdeen	gravis	UK	2009	AUZO00000000	32	-	Persistent sore throat	[5]
ISS 3319	mitis	Italy	1997	JAQO00000000	26	-	Severe pharyngitis/tonsilitis	This study
ISS 4060	gravis	Italy	1999	JAQN00000000	5	-	Severe pharyngitis/tonsilitis	This study
ISS 4746	gravis	Italy	< 2001	JAQP00000000	32	-	Severe pharyngitis/tonsilitis	This study
ISS 4749	gravis	Italy	< 2001	JAQQ00000000	32	-	Severe pharyngitis/tonsilitis	This study

Note: ST designations for strains NCTC 03529 (*atpA*-2, *dnaE*-4, *dnaK*-70, *fusA*-6, *leuA*-7, *odhA*-16, *rpoB*-9) and 31A (*atpA*-2, *dnaE*-2, *dnaK*-New, *fusA*-3, *leuA*-3, *odhA*-2, *rpoB*-3) could not be assigned because they have new combinations of alleles.

References

1. Trost E, Blom J, Soares Sde C, Huang IH, Al-Dilaimi A, et al. (2012) Pangenomic study of *Corynebacterium diphtheriae* that provides insights into the genomic diversity of pathogenic isolates from cases of classical diphtheria, endocarditis, and pneumonia. J Bacteriol 194: 3199-3215.
2. Sangal V, Tucker NP, Burkovski A, Hoskisson PA (2012) The draft genome sequence of *Corynebacterium diphtheriae* bv. mitis NCTC 3529 reveals significant diversity between the primary disease-causing biovars. J Bacteriol 194: 3269.
3. Sangal V, Tucker NP, Burkovski A, Hoskisson PA (2012) Draft genome sequence of *Corynebacterium diphtheriae* biovar intermedius NCTC 5011. J Bacteriol 194: 4738.
4. Cerdeno-Tarraga AM, Efstratiou A, Dover LG, Holden MT, Pallen M, et al. (2003) The complete genome sequence and analysis of *Corynebacterium diphtheriae* NCTC13129. Nucleic Acids Res 31: 6516-6523.
5. Sangal V, Burkovski A, Hunt AC, Edwards B, Blom J, et al. (2014) A lack of genetic basis for biovar differentiation in clinically important *Corynebacterium diphtheriae* from whole genome sequencing. Infect Genet Evol 21: 54-57.
