# Supplementary figures and images for: Adherence and invasive properties of Corynebacterium diphtheriae strains correlates with the predicted membrane-associated and secreted proteome
Source: BMC Genomics. 2015 Oct 9;16:765. doi: 10.1186/s12864-015-1980-8 (PMC4600297; doi:10.1186/s12864-015-1980-8)

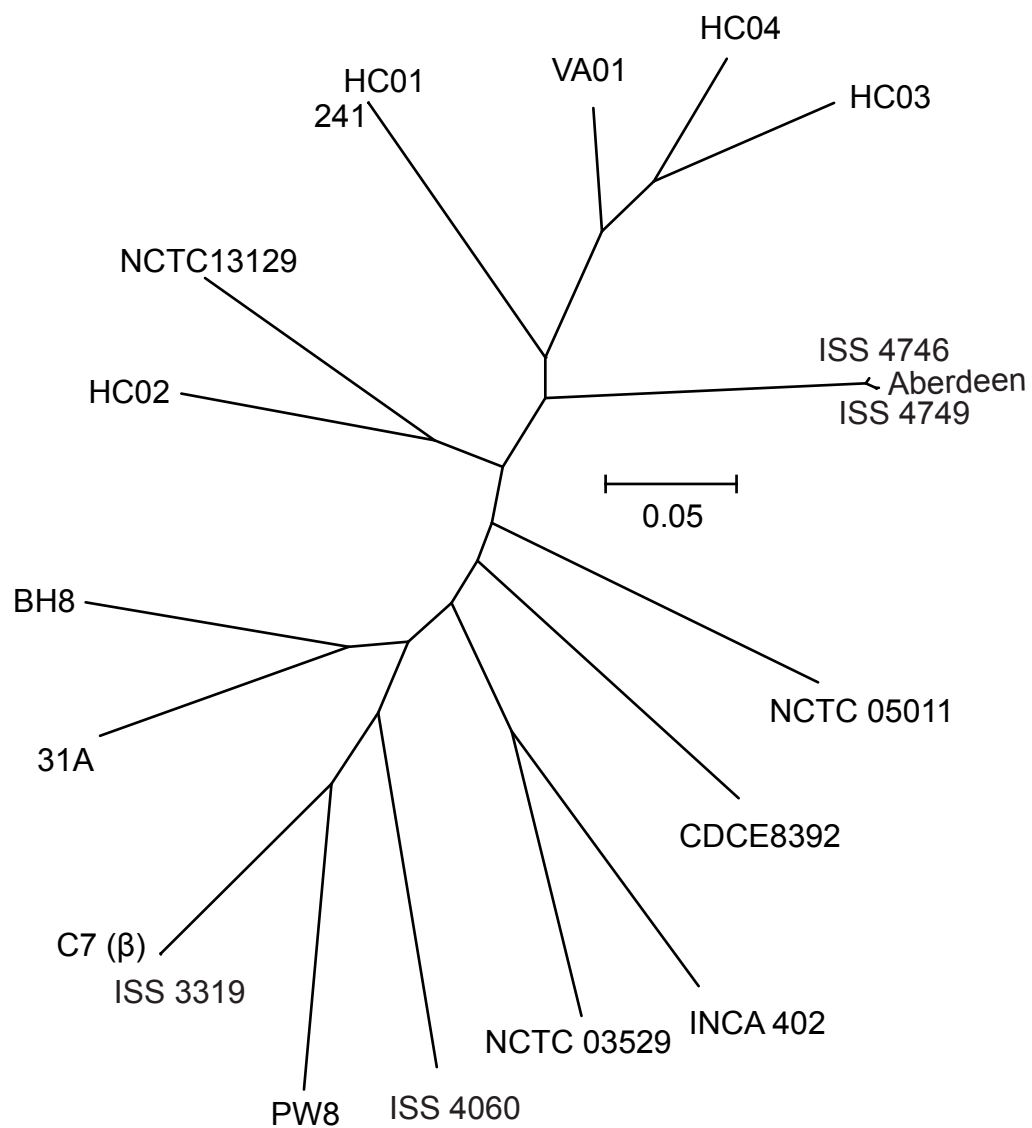

Supplementary Fig. 1. A phylogenetic tree from 400 conserved protein sequences using PhyloPhlAn.

Supplement: Additional file 4: Figure S1. — A phylogenetic tree from a subset of amino acids from 400 universally conserved protein sequences using PhyloPhlAn. (PDF 151 kb) [file 12864_2015_1980_MOESM4_ESM.pdf]
